# Mental health, sleep quality and quality of life in individuals with and without multiple health conditions during home quarantine in India due to the COVID-19 pandemic: a cross-sectional study

**DOI:** 10.12688/f1000research.24321.1

**Published:** 2020-07-17

**Authors:** Ramesh Chandra Patra, Biswajit Kanungo, Parul Bawa

**Affiliations:** 1 Department of Physiotherapy, Lovely Professional University, Jalandhar, Punjab, 144001, India

**Keywords:** COVID-19, Mental Health, Sleep Quality, Quality of Life

## Abstract

**Background:** Since the World Health Organization declared the COVID-19 outbreak a global pandemic, global spread has created several challenges for the general public and health care workers across the world. The primary aim of this study was to assess the psychological stress, sleep quality, and health-related quality of life (QoL) of individuals with multiple health issues during home quarantine caused by the COVID-19 pandemic.

**Methods:** 50 individuals were recruited between 28 ^th^ March and 30 ^th^ April 2020, who have a history of chronic health issues, and 50 individuals with no health issues for this cross-sectional study. Three questionnaires were used to evaluate the mental health [depression anxiety stress scale (DASS-21)], sleep quality [Pittsburgh sleep quality index (PSQI)], and QoL [short form of health-related questionnaire (SF-36)] of participants. Statistical analysis was carried out with Student’s t-test, using SPSS software v16.

**Results:** Baseline demographic characteristics were homogenous for both groups of participants. Intergroup analysis revealed statistically significant differences in mental health (p<0.001), sleep quality (p<0.001), and QoL (p<0.001) between the two groups.

**Conclusion:** Our findings indicate that individuals with chronic health issues exhibit higher mental health problems, lower quality of sleep and have a lower health-related QoL. More research needs to be done for this group of individuals and the Government should plan to care of these individuals.

## Introduction

According to the World Health Organization (WHO), the COVID-19 outbreak is a global pandemic that started at the end of November in China and then gradually spread all over the world ^1, 2^. As neither a treatment nor vaccine have been discovered for this disease, it raises concerns among the public about the spread of infection from confirmed COVID-19 positive cases. The WHO suggests that social isolation helps to limit the growing number of cases of COVID-19, and this has also led to significant fear and anxiety related to the spread of infection in the general public. Excessive fear and apprehension about the spread of infection can lead to acute stress, anxiety, and low quality of sleep ^3, 4^. Many organizations are already working to increase awareness about the societal impact of the on-going pandemic ^5^. For instance, it has been reported that during this pandemic crisis, various factors that impact the health of inviduals, such as prolonged periods of social isolation, fear of unemployment and economic loss, have the potential to increase due to lockdown ^5– 7^. 

The relationship between physical illness and mental health has received increased attention in recent years, and poor mental health is of concern as it may exert a negative effect on an individual's quality of life (QoL) ^8, 9^. Evidence suggests that there is a direct relationship between mental health and sleep quality, a crucial public health issue ^10, 11^. In this study, we aimed to assess the psychological stress, sleep quality, and health-related QoL of individuals with and without multiple health issues during home quarantine in India due to the COVID-19 pandemic.

## Methods

### Ethical considerations

During the conduct of the study, all human ethical principles as per the World Medical Association’s Declaration of Helsinki (2013) and the guidelines of Good Clinical Practice (Indian Council of Medical Research) were observed.

We obtained approval for the study from the Institutional Research Ethics Committee of the Lovely Professional University (LPU), Phagwara, India (LPU/IEC/2020/26/03). All the participants were informed about the procedure of data collection, and a written consent form was obtained from all the participants prior to the study start.

### Study design and setting

This study was an observational cross-sectional study. The study took place at the Department of Physiotherapy, LPU. The study was conducted from 28 ^th^ March to 30 ^th^ April 2020. Full lock down in India was initiated from 28 ^th^ March to 31 ^st^ May 2020. From 31 ^st^ May, the lockdown was extended until 30 ^th^ June for certain containment zones.

### Participant recruitment

A total of 100 participants were recruited for the study. A total of 50 individuals suffering from chronic health issues (Group A; as identified from their clinical records) and 50 individuals with no chronic health issues (Group B) were recruited.

Participants were recruited from individuals undergoing physio treatment at the Department of Physiotherapy, LPU (even though lockdown was ongoing, essential services, such as physio appointments, were ongoing).

Using the convenience sampling method, individuals were contacted via phone to ask if they would like to take part in the study. If the individual consented, in their next physio treatment appointment, they were asked to sign the informed consent form and fill in the three questionnaires (as below).

*Group A inclusion criteria*: clinically pre-diagnosed with hypertension, diabetics, and chronic musculoskeletal conditions.

*Exclusion criteria Groups A and B:* history of any malignancy, recent fracture or trauma, osteoporosis, inflammatory arthritis, and/or cauda equine syndrome.

There were no inclusion criteria for Group B.

### Outcome measures

***Mental health.*** Depression, anxiety, and stress scale (DASS-21) ^12^ was used to assess depression, anxiety and stress. The participants were asked to utilize a four-point severity/frequency scale to show the level of depression, anxiety and stress they were experiencing in the past week. The scale consists of 21 questions with three subscales of depression, stress, and anxiety and each subscale comprises seven questions each. Each subscale comprises of seven statements regarding how the test subject was feeling over the last week and four responses ranging from 0- did not apply to me at all, 1- applied to me some of the time, 2- applied to me for a considerable amount of time to 3- applied to me very much/most of the time. The total score for each subscale gives the severity of that very symptom which has a range from 0 to 21 for each subscale.

***Sleep quality.*** Sleep quality was evaluated through the Pittsburgh Sleep Quality Index (PSQI) ^13^. This index asked participants to answer questions about their sleep habits in the past month. Participants that scored more than 5 were defined as having a low sleep quality.

***Quality of life.*** Health-related QoL was assessed using the MOS 36-item short-form health survey (SF-36) ^14^. The 36 items reflect eight health-related aspects that participants are asked to score, where 100 is defined as perfect health less, and any score less than 100 is defined as poor health.

### Statistical analysis

Baseline characteristics of categorical variables were evaluated using Chi-square test. Quantitative variables were evaluated using Student's t-test, and quantitative variables without normal distribution were measured using the Mann-Whitney U test. Intergroup outcome measures were evaluated through an unpaired t-test. All analyses were carried out on SPSS software v16.

## Results

A total of 110 participants were selected for primary assessment; 10 individuals were excluded as they did not fulfil the inclusion criteria. In total, 50 participants with chronic health issues and 50 without health issues were evaluated for the study.

Demographic characteristics are shown in Table 1. There was no statistical difference between groups for demographic characteristics.

**Table 1.** Demographic characteristics of all patients.

	Group A (n=50)	Group B (n=50)
Age (years, mean)	53.44	52.76
Weight (kg, mean)	65.87	66.45
Height (cm, mean)	163.33	165.33
Gender (%)		
*Female*	70	50
*Male*	30	50
Body mass index (kg/m2, mean)	24.15	23.51
Smoking history (%)		
*No*	82	75
*Yes*	18	25
Marital status (%)		
*Married*	90	85
*Not married*	10	15
Education history (%)		
School level	20	10
*Undergraduate*	60	40
*Postgraduate*	20	50
Socioeconomic status		
*Poor*	0	0
*Average*	60	50
*High*	40	50

Group A, individuals with chronic health issues; Group B, individuals without chronic health issues. Socioeconomic status was calculated as follows: Poor, below Rs 15000/month; average, Rs 15000–100000/month; high, above Rs 100000/month.

**Table 2.** Comparison of outcome measures for Group A and Group B.

Outcome measures		Group A (mean)	Group B (mean)	*Difference*	*P value*
Mental health (DASS-21)	Depression	11.28	5.23	6.05	0.001
	Anxiety	11.14	3.76	7.38	0.001
	Stress	18.58	4.34	14.24	0.001
PSQI	Sleep quality	9.44	5.24	4.00	0.001
Quality of life (SF-36) *	PF	45.21	90.44	45.23	0.001
	RL-PH	25.50	63.51	38.01	0.001
	RL-EH	32.52	79.46	46.94	0.001
	ENG	30.50	84.43	53.93	0.001
	EWB	42.88	83.96	42.08	0.001
	BP	40.00	80.28	40.28	0.001
	GH	35.52	73.80	38.28	0.001

PF: physical functioning, RL-PH: role of limitation-physical health, RL-EH: role of limitation-emotional health, EN: energy, EWB: emotional wellbeing, BP: Body pain, GH: general health, DASS=Depression Anxiety Stress Scale, PSQI= Pittsburgh Sleep Quality Index, *Social life (SL) domain was not considered for this study. Group A, individuals with chronic health issues; Group B, individuals without chronic health issues.

Table 2 presents the scores for Groups A and B for the three outcome measures (mental health, sleep quality and health-related QoL). For all DASS-21 items (mental health), Group A scored higher than Group B, showing higher levels of depression, anxiety and stress in Group A individuals. Similarly, for PSQI, Group A scored higher than Group B, showing poorer sleep quality for Group A individuals. For all SF-26 items, Group A scored lower than Group B, revealing lower health-related QoL in Group A individuals. Unpaired t-tests showed statistically significant differences between the groups for all variables ( *p =* 0.001).

## Discussion

Since the WHO declared the COVID-19 outbreak a global pandemic, many individuals, even those who have not been infected by the virus, are required to follow government rules where it was mandatory to stay at home. In this cross-sectional study, we sought to identify the correlation between chronic health issues and depression, anxiety, stress, quality of sleep and QoL in a population-based study in India during lockdown due to COVID-19. Our findings showed that poor mental health, low sleep quality, and low health-related QoL were higher in individuals with chronic health issues compared with individuals without chronic health issues at the time of home quarantine in India.

Current evidence reveal that COVID-19 causes fear among the Indian population as they are in home quarantined due to lockdown, which can an impact on well-being, increasing the levels of depression, anxiety, stress, reducing sleep quality, and decreasing QoL ^15, 16^.

Considering that lockdown is likely to last for weeks, there is an urgent need to monitor the psycho-physiological well-being of the population and to collect research data to develop evidence-based strategies to reduce negative psychological effects of these unprecedented changes in individuals’ everyday lives ^17, 18^, especially in those with chronic health conditions.

This study has some potential limitations: participants were recruited in only one area of India, we had a small sample size, and only subjective outcome was considered for the study

## Conclusion

The WHO has recommended that individuals with physical and mental disabilities need to take extra care during isolation/quarantine for COVID-19 ^19^. This is supported by the results of this study, which revealed that there is an increased level of depression, anxiety, stress, and reduced sleep quality and QoL shown in individuals suffering from chronic illness compared with individuals without chronic illness during the quarantine period in India.

## Data availability

### Underlying data

Figshare: Mental Health, Sleep Quality and Quality of Life in Subjects with Multiple Health Conditions during Home Quarantine for COVID-19 Pandemic Attack: A Comparison with Healthy Subjects, https://doi.org/10.6084/m9.figshare.12612833 ^20^.

This project contains the following underlying data:

- Group A data
- Group B data

Data are available under the terms of the Creative Commons Zero "No rights reserved" data waiver (CC0 1.0 Public domain dedication).
