# Mental health, sleep quality and quality of life in individuals with and without multiple health conditions during home quarantine in India due to the COVID-19 pandemic: a cross-sectional study

**DOI:** 10.12688/f1000research.24321.2

**Published:** 2022-06-30

**Authors:** Ramesh Chandra Patra, Biswajit Kanungo, Parul Bawa

**Affiliations:** 1 Department of Physiotherapy, Lovely Professional University, Jalandhar, Punjab, 144001, India

**Keywords:** COVID-19, Mental Health, Sleep Quality, Quality of Life

## Abstract

**Background:** Since the World Health Organization declared the COVID-19 outbreak a global pandemic, the global spread has created several challenges for the general public and health care workers across the world. The primary aim of this study was to assess the psychological stress, sleep quality, and health-related quality of life (QoL) of individuals with multiple health issues during home quarantine caused by the COVID-19 pandemic.

**Methods:** 50 individuals were recruited between 28 ^th^ March and 30 ^th^ April 2020, who have a history of chronic health issues, and 50 individuals with no health issues for this cross-sectional study. Three questionnaires were used to evaluate the mental health [depression anxiety stress scale (DASS-21)], sleep quality [Pittsburgh sleep quality index (PSQI)], and QoL [short form of a health-related questionnaire (SF-36)] of participants. Statistical analysis was carried out with Student’s t-test, using SPSS software v16.

**Results:** Baseline demographic characteristics were homogenous for both groups of participants. Intergroup analysis revealed statistically significant differences in mental health (p<0.001), sleep quality (p<0.001), and QoL (p<0.001) between the two groups.

**Conclusion:** Our findings indicate that individuals with chronic health issues exhibit higher mental health problems, lower quality of sleep, and lower health-related QoL. More research needs to be done on this group of individuals, and the Government should plan to care for these individuals.

## Introduction

According to the World Health Organization (WHO), the COVID-19 outbreak is a global pandemic that started at the end of November in China and then gradually spread all over the world ^1, 2^. As neither treatment nor vaccine has been discovered for this disease, it raises concerns among the public about the spread of infection from confirmed COVID-19 positive cases. The WHO suggests that social isolation helps to limit the growing number of cases of COVID-19, and this has also led to significant fear and anxiety related to the spread of infection in the general public. Excessive fear and apprehension about the spread of infection can lead to acute stress, anxiety, and low quality of sleep ^3, 4^. Many organizations are already working to increase awareness about the societal impact of the ongoing pandemic ^5^. For instance, it has been reported that during this pandemic crisis, various factors that impact the health of individuals, such as prolonged periods of social isolation, fear of unemployment, and economic loss, have the potential to increase due to lockdown ^5– 7^. 

The relationship between physical illness and mental health has received increased attention in recent years, and poor mental health is of concern as it may negatively affect an individual’s quality of life (QoL) ^8, 9^. Evidence suggests a direct relationship between mental health and sleep quality, a crucial public health issue ^10, 11^. This study aimed to assess the psychological stress, sleep quality, and health-related QoL of individuals with and without multiple health issues during home quarantine in India due to the COVID-19 pandemic.

## Methods

### Ethical considerations

The study followed all guidelines for ethical conduct per the World Medical Association’s Declaration of Helsinki (2013) and the Good Clinical Practice (Indian Council of Medical Research).

We obtained approval for the study from the Institutional Research Ethics Committee of the Lovely Professional University (LPU), Phagwara, India (LPU/IEC/2020/26/03). All the participants were informed about the data collection procedure, and a written consent form was obtained from all the participants before the study started.

### Study design and setting

This study was an observational cross-sectional study. The study took place at the Department of Physiotherapy, LPU. The study was conducted from 28 ^th^ March to 30 ^th^ April 2020. Full lockdown in India was initiated from 28 ^th^ March to 31 ^st^ May 2020. From 31 ^st^ May, the lockdown was extended until 30 ^th^ June for certain containment zones.

### Participant recruitment

A total of 100 participants were recruited for the study as per their medical history. A total of 50 individuals suffering from chronic health issues (Group A; as identified from their clinical records) and 50 individuals with no chronic health issues (Group B) were recruited.

### Sample size calculation

Sample size was calculated using G power software 3.1. The estimated sample size calculated for the study was 51 (95% confidence interval, power of the study was 80% where the effect size was considered as 0.5).

Individuals undergoing physio treatment at the Department of Physiotherapy, LPU (even though lockdown was going on, essential services, such as physio appointments, were continuing) were recruited for the study.

Individuals were contacted via phone to ask if they would like to take part in the study. As they were supposed to take physiotherapy services from our Organization, so before starting the treatment session, they were requested to sign the consent form, fill the questionnaire and were informed about the study procedure.

*Group A inclusion criteria*: Individuals clinically pre-diagnosed with hypertension, diabetics, and chronic musculoskeletal conditions were included in this group.

Group B inclusion criteria: Individuals with no chronic health issues were included in this group.

*Groups A and B exclusion criteria:* Individuals with history of any malignancy, recent fracture or trauma, osteoporosis, inflammatory arthritis, and/or cauda equine syndrome were excluded from this study.

### Outcome measures

***Mental health.*** Depression, anxiety, and stress scale (DASS-21) ^12^ was used to assess depression, anxiety, and stress. The participants were asked to utilize a four-point severity/frequency scale to show the level of depression, anxiety, and stress they were experiencing in the past week.

***Sleep quality.*** Sleep quality was evaluated through the Pittsburgh Sleep Quality Index (PSQI) ^13^. This index asked participants to answer questions about their sleep habits in the past month. Participants that scored more than 5 were defined as having a low sleep quality.

***Quality of life.*** Health-related QoL was assessed using the MOS 36-item short-form health survey (SF-36) ^14^. The 36 items reflect eight health-related aspects that participants are asked to score, where 100 is defined as perfect health less, and any score less than 100 is defined as poor health.

### Statistical analysis

Baseline characteristics of categorical variables were evaluated using the Chi-square test. Quantitative variables were evaluated using Student’s t-test, and quantitative variables without normal distribution were measured using the Mann-Whitney U test. Intergroup outcome measures were evaluated through an unpaired t-test. All analyses were carried out on SPSS software v16.

## Results

A total of 110 participants were selected for primary assessment; 10 individuals were excluded as they did not fulfill the inclusion criteria. In total, 50 participants with chronic health issues and 50 without health issues were evaluated for the study.

Demographic characteristics are shown in Table 1. There was no statistical difference between groups for demographic characteristics.

**Table 1.** Demographic characteristics of all patients.

	Group A (n=50)	Group B (n=50)
Age (years, mean)	53.44	52.76
Weight (kg, mean)	65.87	66.45
Height (cm, mean)	163.33	165.33
Gender (%)		
*Female*	70	50
*Male*	30	50
Body mass index (kg/m2, mean)	24.15	23.51
Smoking history (%)		
*No*	82	75
*Yes*	18	25
Marital status (%)		
*Married*	90	85
*Not married*	10	15
Education history (%)		
School level	20	10
*Undergraduate*	60	40
*Postgraduate*	20	50
Socioeconomic status		
*Poor*	0	0
*Average*	60	50
*High*	40	50

Group A, individuals with chronic health issues; Group B, individuals without chronic health issues. Socioeconomic status was calculated as follows: Poor, below Rs 15000/month; average, Rs 15000–100000/month; high, above Rs 100000/month.

Table 2 presents Groups A and B scores for the three outcome measures (mental health, sleep quality, and health-related QoL). For all DASS-21 items (mental health), Group A scored higher than Group B, showing higher levels of depression, anxiety, and stress in Group A individuals. Similarly, for PSQI, Group A scored higher than Group B, showing poorer sleep quality for Group A individuals. For all SF-26 items, Group A scored lower than Group B, revealing lower health-related QoL in Group A individuals. Unpaired t-tests showed statistically significant differences between the groups for all variables ( *p =* 0.001).

**Table 2.** Comparison of outcome measures for Group A and Group B.

Outcome measures		Group A (mean)	Group B (mean)	*Difference*	*P value*
Mental health (DASS-21)	Depression	11.28	5.23	6.05	0.001
	Anxiety	11.14	3.76	7.38	0.001
	Stress	18.58	4.34	14.24	0.001
PSQI	Sleep quality	9.44	5.24	4.00	0.001
Quality of life (SF-36) *	PF	45.21	90.44	45.23	0.001
	RL-PH	25.50	63.51	38.01	0.001
	RL-EH	32.52	79.46	46.94	0.001
	ENG	30.50	84.43	53.93	0.001
	EWB	42.88	83.96	42.08	0.001
	BP	40.00	80.28	40.28	0.001
	GH	35.52	73.80	38.28	0.001

PF: physical functioning, RL-PH: role of limitation-physical health, RL-EH: role of limitation-emotional health, EN: energy, EWB: emotional wellbeing, BP: Body pain, GH: general health, DASS=Depression Anxiety Stress Scale, PSQI= Pittsburgh Sleep Quality Index, *Social life (SL) domain was not considered for this study. Group A, individuals with chronic health issues; Group B, individuals without chronic health issues.

## Discussion

Since the WHO declared the COVID-19 outbreak a global pandemic, many individuals, even those who have not been infected by the virus, are required to follow government rules where it was mandatory to stay at home. In this cross-sectional study, we sought to identify the correlation between chronic health issues and depression, anxiety, stress, quality of sleep and QoL in a population-based study in India during lockdown due to COVID-19. Our findings showed that poor mental health, low sleep quality, and low health-related QoL were higher in individuals with chronic health issues compared with individuals without chronic health issues at the time of home quarantine in India.

Current evidence reveals that COVID-19 causes fear among the Indian population as they are at home quarantined due to lockdown, which can impact wellbeing, increasing depression, anxiety, stress, reducing sleep quality, and decreasing sleep QoL ^15, 16^.

Considering that the lockdown is likely to last for weeks, there is an urgent need to monitor the psycho-physiological wellbeing of the population and to collect research data to develop evidence-based strategies to reduce the negative psychological effects of these unprecedented changes in individuals’ everyday lives ^17, 18^, especially in those with chronic health conditions.

This study has some potential limitations: participants were recruited in only one area of India, we had a small sample size, and the only subjective outcome was considered for the study.

### Limitations

There are inherent limitations to this study. A major limitation of this study was the small sample size. Another limitation of the study was the involvement of only a single medical center. Additional studies from multiple centers’ are warranted.

### Recommendations

The present study substantiates that individuals with chronic illness have increased levels of depression, anxiety, stress, reduced sleep quality, and QoL compared with individuals without chronic illness during the quarantine period in India. Therefore, it is recommended that these individuals be constantly monitored for their psychological wellbeing during quarantine. To further substantiate the research, large comprehensive multi-centric studies should be conducted.

## Conclusion

The WHO has recommended that individuals with physical and mental disabilities need to take extra care during isolation/quarantine for COVID-19 ^19^. This is supported by the results of this study, which revealed an increased level of depression, anxiety, stress, and reduced sleep quality. QoL has been shown in individuals suffering from a chronic illness compared with those without chronic illness during the quarantine period in India.

## Data availability

### Underlying data

Figshare: Mental Health, Sleep Quality and Quality of Life in Subjects with Multiple Health Conditions during Home Quarantine for COVID-19 Pandemic Attack: A Comparison with Healthy Subjects, https://doi.org/10.6084/m9.figshare.12612833 ^20^.

This project contains the following underlying data:

- Group A data
- Group B data

Data are available under the terms of the Creative Commons Zero "No rights reserved" data waiver (CC0 1.0 Public domain dedication).
