# Supplementary material for: Mental health, sleep quality and quality of life in individuals with and without multiple health conditions during home quarantine in India due to the COVID-19 pandemic: a cross-sectional study
Source: F1000Res. 2024 Jul 29;9:718. Originally published 2020 Jul 17. [Version 3] doi: 10.12688/f1000research.24321.3 (PMC11795741; doi:10.12688/f1000research.24321.3)
Supplement: Peer review history

# Reviewer report: Reviewer response for version 3

**DOI:** 10.5256/f1000research.169415.r353430

**Published:** 2024-12-30

**Authors:** Isabell Wagenhäuser

**Affiliations:** 1 University Hospital Würzburg, Würzburg, Germany

At the beginning of the COVID-19 pandemic in India, the work presented examines the psychological effects of the lockdown on people with chronic illnesses in a controlled study compared to healthy people.  

 - Since the lockdown conditions were very different from country to country, it would be good to better define what lockdown meant in India in the introduction.

 - Please add a clear definition of the inclusion criteria in the methods.

 - Table 1: Are groups A and B comparable? If there are differences, this must be interpreted and taken into account statistically. Please compare the items statistically and add p-values.

 - The results of the study are extremely brief and incomplete.

 - Even if the groups are relatively comparable, statistical consideration of the various factors must be added -> what factors influence the occurrence of sleep disorders, depression, etc. (regression analysis)? 

 - Please add to the LImitations that unbalanced gender ratio, only short period at the beginning of the pandemic. It is unclear what the situation will be in a second lockdown, e.g. in winter 2020/21, etc. 

 - Please also discuss whether there is also literature on whether COVID-19 vaccination or infection can cause depression / sleep disorders and whether the lockdown is worth it in terms of mental risk. (e.g. Wagenhäuser I, et al., 2024 [Ref 1] or Athanasiou N, et al., 2023 [Ref 2])

Is the work clearly and accurately presented and does it cite the current literature?

Partly

If applicable, is the statistical analysis and its interpretation appropriate?

Yes

Are all the source data underlying the results available to ensure full reproducibility?

Partly

Is the study design appropriate and is the work technically sound?

Partly

Are the conclusions drawn adequately supported by the results?

Yes

Are sufficient details of methods and analysis provided to allow replication by others?

Partly

Reviewer Expertise:

Epidemiology, immunology and diagnostics of acute respiratory infections

I confirm that I have read this submission and believe that I have an appropriate level of expertise to confirm that it is of an acceptable scientific standard, however I have significant reservations, as outlined above.

## References

[1] : The relationship between mental health, sleep quality and the immunogenicity of COVID-19 vaccinations. *J Sleep Res* .2024;33(3) : 10.1111/jsr.13929 e13929 10.1111/jsr.13929 37177872

[2] : Association of sleep duration and quality with immunological response after vaccination against severe acute respiratory syndrome coronavirus-2 infection. *J Sleep Res* .2023;32(1) : 10.1111/jsr.13656 e13656 10.1111/jsr.13656 35670298 PMC9348328

# Reviewer report: Reviewer response for version 3

**DOI:** 10.5256/f1000research.169415.r308385

**Published:** 2024-08-16

**Authors:** Uzma Zaidi

**Affiliations:** 1 Princess Nourah bint Abdulrahman University, Riyadh, Saudi Arabia

The authors conducted the study on the initial stage of COVID-19; therefore, the significance is undeniable. Moreover, the exploration of psychological or psychologically related factors of QoL and sleep, besides health conditions is a very valuable aspect. The result allows for the recommendation of more comprehensive policymaking for people who are suffering from multiple health conditions. The authors have improved the quality of the manuscript by responding aptly to the comments of reviewers.  However, some improvement or explanation is still required for the methodology, statistical analysis, and results section.

**Abstract:**

 The conclusion needs some improvement.

 “Our findings indicate that individuals with chronic health ….. More research is required and also the government should plan on taking care of those patients.”

Suggestion: “Our findings indicate that individuals with chronic health issues exhibit higher mental health problems, lower quality of sleep, and have a lower health-related QoL during the COVID-19 pandemic. More research is required and also the government should plan policies for patients with multiple health conditions.”

**Methods:**

**Outcome measure:**

 Please correct and update the description of the scoring method for MOS 36-item health-related survey. Therefore, the replication of the method will be authentic and easy.

 “The 36 items reflect …….., where 100 is defined as perfect health less, and any score less than 100 is defined as poor health.”

Suggestion: I am sharing the description along with the citation.

 The scoring is from 0-100. Higher scores indicate better health status, whereas a low score presents poor health. The mean score of 50 has been considered as a cutoff score for all scales.[1]

**Statistical Analysis:**

Suggestion: In Table 1 add a column presenting the calculation of Chi-square to present statistical difference.

**Results:**

Suggestion: As researchers, it is wise to report the results in a more precise manner. According to the standardized scoring categories of DASS, mean scores of anxiety and stress of group A fall at an extremely severe level, whereas depression scores are falling in the range of severe levels. The relevant categories of DASS can be discussed in the result description for group B. It will strengthen the study findings. As in the normal population mean score of depression and anxiety is falling within the mild level. For more details check scoring categories: https://www.healthfocuspsychology.com.au/tools/dass-21/

**Discussion:**

Suggestion: There is another very interesting result related to QoL that needs to be discussed. The highest mean score in group A is in the physical functioning (PF) domain. As all the participants were receiving physiotherapy that could be one of the reasons. Authors can address it better. The lowest mean in both of the groups can be seen in the Role of the limitation-physical health (RL-PH) domain.

Is the work clearly and accurately presented and does it cite the current literature?

Yes

If applicable, is the statistical analysis and its interpretation appropriate?

Partly

Are all the source data underlying the results available to ensure full reproducibility?

Yes

Is the study design appropriate and is the work technically sound?

Yes

Are the conclusions drawn adequately supported by the results?

Yes

Are sufficient details of methods and analysis provided to allow replication by others?

Partly

Reviewer Expertise:

Clinical Psychology, Health Psychology, Personality Psychology, Mental Illness, Neuropsychology, Organizational Behavior

I confirm that I have read this submission and believe that I have an appropriate level of expertise to confirm that it is of an acceptable scientific standard, however I have significant reservations, as outlined above.

## References

[1] : SF-36 health survey update. *Spine (Phila Pa 1976)* .2000;25(24) : 10.1097/00007632-200012150-00008 3130-9 10.1097/00007632-200012150-00008 11124729

# Reviewer report: Reviewer response for version 2

**DOI:** 10.5256/f1000research.80174.r167559

**Published:** 2023-04-21

**Authors:** Uzma Zaidi

**Affiliations:** 1 Princess Nourah bint Abdulrahman University, Riyadh, Saudi Arabia

The authors conducted the study on the initial stage of COVID-19; therefore, the significance is undeniable. Moreover, the exploration of psychological or psychologically related factors of QoL and sleep, besides health conditions is a very valuable aspect. The result allows for the recommendation of more comprehensive policy making for people who are suffering from multiple health conditions. The authors have improved the quality of the manuscript by responding aptly to the comments of reviewers. However, some improvement or explanation is still required for the methodology, statistical analysis, and results section.

**Abstract:**

 The conclusion needs some improvement.

 “Our findings indicate that individuals with chronic health ….. More research is required and also the government should plan on taking care of those patients.”

Suggestion: “Our findings indicate that individuals with chronic health ...... More research is required and also the government should plan policies for patients with multiple health conditions.”

**Methods:**

**Outcome measure:**

 Please correct and update the description of the scoring method for MOS 36-item health-related survey. Therefore, the replication of the method will be authentic and easy.

 “The 36 items reflect …….., where 100 is defined as perfect health less, and any score less than 100 is defined as poor health.”

Suggestion: I am sharing the description along with the citation.

 The scoring is from 0-100. Higher scores indicate better health status, whereas a low score presents poor health. The mean score of 50 has been considered as a cutoff score for all scales ^1^.

**Statistical Analysis:**

Suggestion: In Table 1 add a column presenting the calculation of Chi-square.

 Add a source for socioeconomic slabs in the footnote of Table 1.

 Authors are reporting that “Quantitative variables were evaluated using Student’s t-test, and quantitative variables without normal distribution were measured using the Mann-Whitney U test.” However, table 2 does not present the student t-test and Mann-Whitney U test explicitly.

Suggestion: Provide an SD column for both groups, remove the difference column, and add a column for 't' or 'U' before the P value.

**Results:**

Suggestion: As researchers, it is wise to report the results in a more precise manner. According to the standardized scoring categories of DASS, mean scores of anxiety and stress of group A fall at an extremely severe level, whereas depression scores are falling in the range of severity levels. The relevant categories of DASS can be discussed in the result description for group B. It will strengthen the study findings. As in the normal population mean score of depression and anxiety is falling within the mild level. For more details, check scoring categories.

**Discussion:**

Suggestion: There is another very interesting result related QoL that needs to be discussed. The highest mean score in group A is in the physical functioning (PF) domain. As all the participants were receiving physiotherapy that could be one of the reasons. Authors can address it better. The lowest mean in both of the groups can be seen in the Role of the limitation-physical health (RL-PH) domain.

Is the work clearly and accurately presented and does it cite the current literature?

Yes

If applicable, is the statistical analysis and its interpretation appropriate?

Partly

Are all the source data underlying the results available to ensure full reproducibility?

Yes

Is the study design appropriate and is the work technically sound?

Yes

Are the conclusions drawn adequately supported by the results?

Yes

Are sufficient details of methods and analysis provided to allow replication by others?

Partly

Reviewer Expertise:

Clinical Psychology, Health Psychology, Personality Psychology, Mental Illness, Neuropsychology, Organizational Behavior

I confirm that I have read this submission and believe that I have an appropriate level of expertise to confirm that it is of an acceptable scientific standard, however I have significant reservations, as outlined above.

## References

[1] : SF-36 health survey update. *Spine (Phila Pa 1976)* .2000;25(24) : 10.1097/00007632-200012150-00008 3130-9 10.1097/00007632-200012150-00008 11124729

# Reviewer report: Reviewer response for version 1

**DOI:** 10.5256/f1000research.26834.r82045

**Published:** 2021-04-08

**Authors:** Kanwar Hamza Shuja

**Affiliations:** 1 Capital University of Science and Technology (CUST), Islamabad, Pakistan

Firstly thank you for giving me the chance for reviewing the article. It's a well written article though there are places where some sentences needs to be rewritten and checked for grammar. However, my biggest concern with this article is regarding its sample size. My first question is how did authors come up with a sample size of 50? Did they selected it themselves or what was the reason behind it or did they used sampling size calculator. Because a sample of 50 is too low for concluding this study.

 Secondly, again with the sample but this time inclusion criteria for the group B was not set by the authors. Having no inclusion criteria could lead to potentially adding participants who may also be medically ill, or were these participants were inquired prior during recruitment if they are medically sound. And if they were asked that would have become their inclusion criteria. But unfortunately, I was unable to find any such thing in demographic information.

 Third the instruments used are for measuring psychological variables in general. What I mean by this is that nowhere was I able to find anything where the participants were asked their thoughts on Covid-19 or its effect on them. Now depression, stress or anxiety could be potentially due to multiple factors same with the reasons for disturbed quality of life or sleep. The authors should have added some questions in demographic which could help the participants and the readers to know that the findings here are actually due to Covid-19 and not due to any external factor.

 Fourth it would would be nice if the authors can add limitations and recommendations heading before conclusion.

 Thank you and good luck.

Is the work clearly and accurately presented and does it cite the current literature?

Yes

If applicable, is the statistical analysis and its interpretation appropriate?

Yes

Are all the source data underlying the results available to ensure full reproducibility?

Yes

Is the study design appropriate and is the work technically sound?

Yes

Are the conclusions drawn adequately supported by the results?

Yes

Are sufficient details of methods and analysis provided to allow replication by others?

Partly

Reviewer Expertise:

Public Health, Criminal Psychology, Clinical Psychology.

I confirm that I have read this submission and believe that I have an appropriate level of expertise to state that I do not consider it to be of an acceptable scientific standard, for reasons outlined above.

# Response

**Published:** 2022-06-16

**Authors:** Ramesh Patra

**Affiliations:** Lovely Professional University, India

First, we would like to thank the honourable reviewers and editor for their constructive criticisms and suggestive comments, which were of great help in improving the quality and clarity of the current manuscript. We have read each reviewer’s comments carefully and, accordingly, made utmost efforts to address each comment point-by-point. We much appreciate the reviewer’s time, effort, and detailed explanations. We believe that the revised version of our manuscript is now more focused, scientific, understandable, and better arranged. All changes are highlighted with red color.

 Reviewer Comment:

 Query 1:

 Firstly thank you for giving me the chance for reviewing the article. It's a well written article though there are places where some sentences needs to be rewritten and checked for grammar.

 Response:

 Thank you for your valuable comments. We have modified the text with proper grammar usage as per your suggestions.

 Query 2:

 However, my biggest concern with this article is regarding its sample size. My first question is how did authors come up with a sample size of 50? Did they selected it themselves or what was the reason behind it or did they used sampling size calculator. Because a sample of 50 is too low for concluding this study.

 Response:

 The total sample taken was 100. We have calculated the sample size using G power software 3.1 which was found to be around 51 (95% confidence interval, power of the study was 80% where the effect size was considered as 0.5). The sample size calculation has been added in the revised manuscript under the method section.

 Query 3:

 Secondly, again with the sample but this time inclusion criteria for the group B was not set by the authors. Having no inclusion criteria could lead to potentially adding participants who may also be medically ill, or were these participants inquired prior during recruitment if they are medically sound. And if they were asked that would have become their inclusion criteria. But unfortunately, I was unable to find any such thing in demographic information.

 Response:

 As suggested, we have included the inclusion criteria for Group B.

 Query 4:

 Third, the instruments used, are for measuring psychological variables in general. What I mean by this is that nowhere was I able to find anything where the participants were asked their thoughts on Covid-19 or its effect on them. Now depression, stress or anxiety could be potentially due to multiple factors same with the reasons for disturbed quality of life or sleep. The authors should have added some questions in demographic which could help the participants and the readers to know that the findings here are actually due to Covid-19 and not due to any external factor.

 Response:

 We have used the following tools to measure all the three measure outcomes.

1. DASS-21 tool was used to measure the mental health.
2. PSQI was used to measure sleep quality.
3. MOS-36 was used to measure the health-related QoL.

We have mentioned the tools in the methodology section and also given references.

 Query 5:

 Fourth it would be nice if the authors can add limitations and recommendations heading before conclusion.

 Response: We have included the limitations and recommendations as per your suggestions.

# Reviewer report: Reviewer response for version 1

**DOI:** 10.5256/f1000research.26834.r73792

**Published:** 2020-11-13

**Authors:** Shyamal Koley

**Affiliations:** 1 Department of Physiotherapy, Guru Nanak Dev University, Amritsar, India

The Abstract, Introduction, Methods, Results, & Discussion Portions of the article are written nicely. The only correction needed is in Acknowledgments where "  The author would......" should be written as " The authors would......".

 References portion are written as per the style of the journal and adequate and expressive.

 The article is timely written and carries sufficient applicability.

**Detailed Report:**

 COVID-19 is a serious global pandemic which affects physical, mental, social and societal aspects of individuals of all strata of the society. The present study attempted justly in this regard. It is a very recent complication, thus not much literature would be available. Considering the above fact, the literature cited in the article was justified. It was quite difficult to collect the samples in the time of Lockdown prevailed then nationwide. Still the work would be considered as technically sound. In such type of study, the controls are not necessarily required, pre- and post-conditions may work. The methods and analyses were sound with proper replicability. Though the sample size was small (n=100), statistical analyses (using Chi-square test, Student’s t-test and Man-Whitney U test) were appropriate. The conclusion drawn in the study was adequately supported by the results. Small sample size was one of the limitations of the study.

Is the work clearly and accurately presented and does it cite the current literature?

Yes

If applicable, is the statistical analysis and its interpretation appropriate?

Yes

Are all the source data underlying the results available to ensure full reproducibility?

Yes

Is the study design appropriate and is the work technically sound?

Yes

Are the conclusions drawn adequately supported by the results?

Yes

Are sufficient details of methods and analysis provided to allow replication by others?

Yes

Reviewer Expertise:

Kinanthropometry, Biomechanics, Public Health & Lectinology.

I confirm that I have read this submission and believe that I have an appropriate level of expertise to confirm that it is of an acceptable scientific standard.

# Response

**Published:** 2020-11-13

**Authors:** Ramesh Patra

**Affiliations:** Lovely Professional University, India

Thank You so much for your Valuable response and comments.
